# Avoiding Hemodynamic Collapse During Induction of General Anesthesia in a Patient With Unrecognized Cardiac Tamponade: A Case Report

**DOI:** 10.1002/ccr3.73879

**Published:** 2026-11-05

**Authors:** Itsuki Suganuma, Tomoo Yuba, Katsunao Tanaka, Yu Matsumoto, Akira Iura, Takeshi Yoshida

**Affiliations:** ^1^ Department of Anesthesiology and Intensive Care Medicine The University of Osaka Graduate School of Medicine Osaka Japan; ^2^ Department of Anesthesiology Kansai Rosai Hospital Amagasaki Japan; ^3^ Department of Cardiovascular Medicine The University of Osaka Graduate School of Medicine Osaka Japan

**Keywords:** cardiac tamponade, case report, general anesthesia, pericardial drainage, pericardial effusion

## Abstract

Emergency anesthesia requests may underestimate evolving cardiovascular pathology. When bedside findings are discordant with the initial working diagnosis, anesthesiologists should defer induction, reassess independently, and escalate multidisciplinary care before initiating general anesthesia.

**Abbreviations**
A‐line: arterial line
BP: blood pressure
CT: computed tomography
OR: operating room
PI: perfusion index
POCUS: point‐of‐care ultrasound
SpO_2_
: peripheral oxygen saturation
TTE: transthoracic echocardiography

## 1 Introduction

The hemodynamics of a patient with cardiac tamponade can deteriorate abruptly during the induction of general anesthesia because anesthetics and positive‐pressure ventilation may reduce sympathetic tone, venous return, and cardiac filling [1, 2]. When tamponade physiology is suspected, stabilization before induction—including pericardial drainage under local anesthesia when feasible—may prevent catastrophic hemodynamic collapse [3]. We report an emergency case in which rapid reassessment in the operating room prompted pre‐induction pericardial drainage, enabling the safe induction of general anesthesia. Written informed consent was obtained from the patient for publication of this case report and accompanying images.

## 2 Case History/Examination

A 50‐year‐old Japanese man presented with a medical history significant for bladder cancer (status post robot‐assisted radical cystectomy), left lung metastasis, a pericardial effusion, a left pleural effusion, type 2 diabetes mellitus, and a previous COVID‐19 infection. He was emergently referred by the urology team for a pleurostomy (thoracic window) to alleviate worsening dyspnea. Given the long‐standing pleural effusion and anticipated septation and loculation, simple thoracentesis or tube drainage alone was considered unlikely to achieve sufficient and durable decompression; therefore, the procedure was planned under general anesthesia. The initial emergency consultation cited respiratory compromise attributed mainly to a progressive pleural effusion and the need for prompt operative intervention. Although a pericardial effusion had been documented on prior imaging, its potential hemodynamic significance was not initially highlighted to the anesthesia team during the consultation. Several days earlier, a cardiology consultation was obtained following a chest computed tomography (CT) scan. Bedside echocardiography performed at that time revealed a pericardial effusion; however, the amount and distribution of the effusion were considered unfavorable for percutaneous drainage because a safe subxiphoid approach was not feasible. Consequently, conservative management was chosen after weighing the risks and benefits. On the day of surgery, no repeat echocardiographic assessment was performed before transferring the patient to the operating room. The patient was therefore transferred with a working diagnosis attributing his dyspnea primarily to a pleural effusion and a recent COVID‐19 infection. At this time, the hemodynamic significance of the progressive pericardial effusion remained unappreciated.

Although preparing the operating room and reviewing the available clinical information, the attending anesthesiologist noted the presence of the pericardial effusion on prior imaging. By that time, the patient had already arrived in the operating room. Upon arrival, he exhibited tachycardia and tachypnea and was unable to tolerate the supine position. He also exhibited diaphoresis, cool extremities, and a markedly decreased perfusion index (PI) on pulse oximetry. The attending anesthesiologist then re‐evaluated the serial preoperative chest CT images and confirmed that the pericardial effusion had progressively enlarged. Concern for a pre‐induction tamponade was driven by the temporal increase in the pericardial effusion on CT and the patient's shock‐like clinical presentation upon arrival in the operating room (severe orthopnea, diaphoresis, cool extremities, and a markedly decreased PI). Representative preoperative chest CT images demonstrating a circumferential pericardial effusion are shown in Figure 1. Consequently, an arterial line was placed before induction to facilitate continuous hemodynamic monitoring. The arterial pressure waveform demonstrated marked respiratory variation, with an inspiratory decrease in systolic arterial pressure of approximately 20 mmHg, consistent with pulsus paradoxus.

**FIGURE 1:**
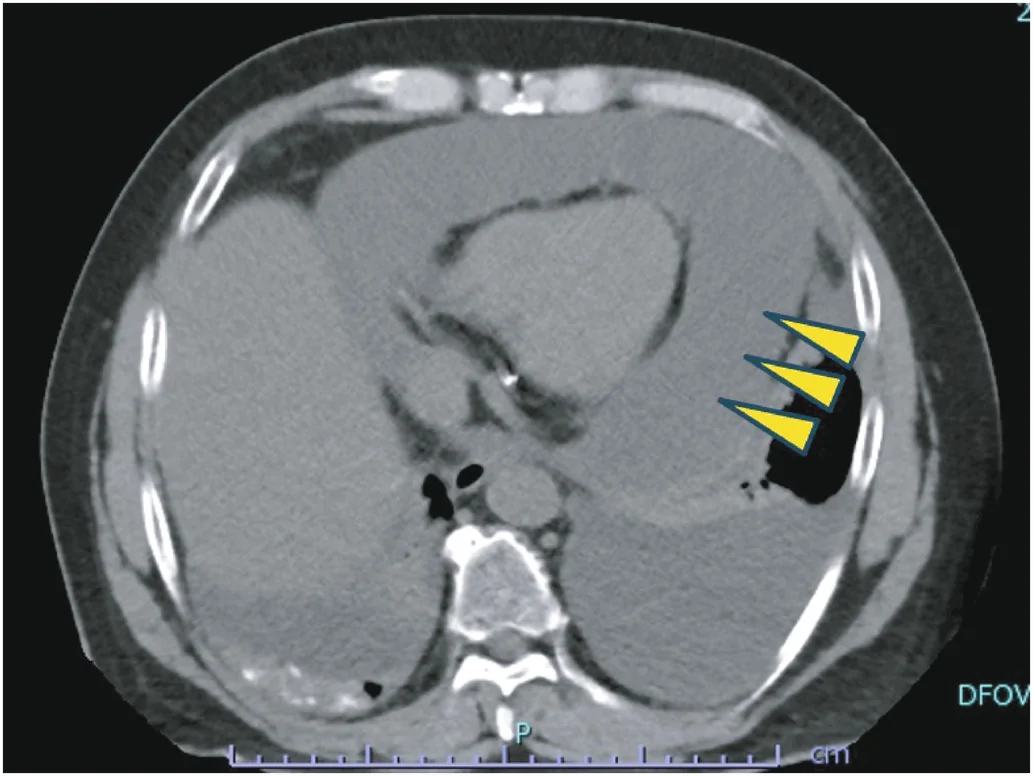
Preoperative chest computed tomography demonstrating a progressive circumferential pericardial effusion. Axial chest computed tomography image obtained before surgery showing a circumferential pericardial effusion; in conjunction with the clinical findings, this raised clinical concern for an evolving tamponade physiology. The volume of pericardial fluid had increased compared with prior imaging, suggesting the progression of the effusion (yellow arrowheads).

## 3 Methods (Differential Diagnosis, Investigations and Treatment)

We deferred induction to perform an immediate reassessment and escalate care. Because the pleurostomy was required on an emergency basis to relieve respiratory compromise, postponing the surgery without prior stabilization was considered unsafe.

Taken together, these findings raised strong concern for evolving tamponade physiology due to the expanding pericardial effusion, with impaired cardiac filling and a circulation likely dependent on compensatory tachycardia and sympathetic tone. We therefore considered that attenuation of sympathetic tone and venous return during induction of general anesthesia could precipitate hemodynamic collapse. Consequently, we determined that, if feasible, pericardial drainage under local anesthesia should precede induction, and we requested an urgent multidisciplinary consultation (with cardiology and cardiovascular surgery). The cardiovascular surgery team deemed the procedure feasible and performed pericardial drainage under local anesthesia prior to induction. Approximately 1000 mL of hemorrhagic pericardial fluid was evacuated. Histopathological examination of the pericardial tissue revealed metastatic bladder cancer, supporting the diagnosis of malignant pericardial effusion. Hemodynamic variables immediately before and after pericardial drainage, including heart rate, respiratory variation in the arterial pressure waveform, and PI, are summarized in Table 1.

**TABLE 1:** Hemodynamic variables before and after the pericardial drainage procedure.

	Drainage	
	Before	After
Respiratory rate (breaths/min)	28	17
Heart rate (bpm)	111	95
Blood pressure (mmHg)	90/59	111/49
Respiratory variation in the arterial pressure waveform (change in systolic pressure)	Marked (20 mmHg)	Diminished (9 mmHg)
Perfusion index (PI, %)	0.42	1.24

*Note:* The table presents the patient's respiratory rate, heart rate, arterial blood pressure, respiratory variation in the arterial pressure waveform (expressed as the difference between the maximal and minimal systolic pressures within a respiratory cycle), and PI immediately before and after the pericardial drainage procedure under local anesthesia. PI was measured via pulse oximetry and reflects peripheral perfusion.

## 4 Conclusions and Results (Outcome and Follow‐Up)

Immediately after pericardial drainage, the patient's heart rate decreased, systolic arterial pressure and pulse pressure increased, respiratory variation in systolic arterial pressure diminished, and the PI increased substantially. Based on these changes, we determined that the risk of circulatory collapse secondary to sympathetic suppression had been substantially reduced. Consequently, general anesthesia was uneventfully induced with remifentanil and remimazolam, and neuromuscular blockade was achieved with rocuronium. The patient's trachea was intubated orally, and positive‐pressure ventilation was initiated. During induction and immediately after the initiation of mechanical ventilation, the patient's blood pressure remained stable without clinically significant hypotension. Vasopressors were prepared in anticipation of potential hemodynamic instability; however, they were not required. At the conclusion of the surgery, the patient awakened promptly in the operating room and was extubated uneventfully. Thereafter, both his respiratory and hemodynamic statuses remained stable with supplemental oxygen administered via a face mask at 6 L/min. He was transferred to the general ward without immediate postoperative complications. The time course of the patient's heart rate, arterial pressure, and PI during the peri‐procedural period of the pericardial drainage, extracted from the anesthetic record, is illustrated in Figure 2.

**FIGURE 2:**
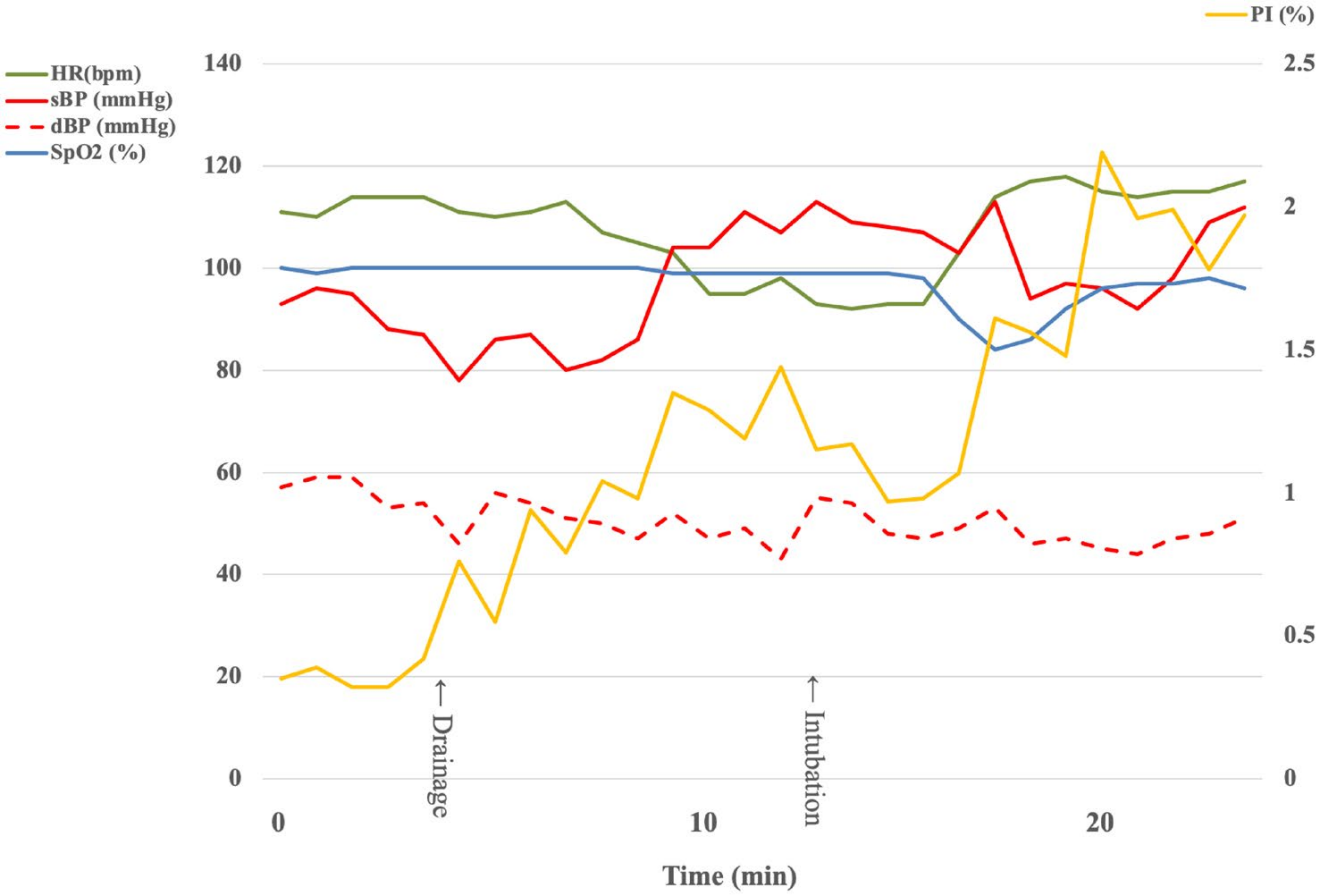
Time course of the patient's heart rate, arterial pressure, oxygen saturation, and PI during the peri‐procedural period of the pericardial drainage. Trends in the patient's heart rate, invasive arterial blood pressure, oxygen saturation (SpO_2_), and perfusion index (PI) were extracted from the anesthetic record before and after the pericardial drainage procedure under local anesthesia. The timings of the pericardial drainage and the tracheal intubation are indicated; following these interventions, the systolic arterial pressure and PI increased while the heart rate decreased, reflecting hemodynamic stabilization.

## 5 Discussion

Cardiac tamponade is a hemodynamic condition in which increased intrapericardial pressure impairs diastolic cardiac filling, resulting in reduced stroke volume and cardiac output. Compensatory tachycardia and sympathetic activation may initially maintain systemic perfusion, while exaggerated ventricular interdependence can produce pulsus paradoxus. In the present case, the progressive pericardial effusion, shock‐like clinical findings, and an inspiratory decrease in systolic arterial pressure of approximately 20 mmHg collectively supported evolving tamponade physiology.

This case illustrates a vulnerable point in emergency perioperative workflows: a patient may arrive in the operating room with an evolving cardiovascular condition that was not fully appreciated during the initial surgical consultation. The initial clinical focus in this case was the respiratory compromise requiring an urgent pleurostomy; however, the patient's clinical presentation upon arrival was not fully explained by an isolated pleural effusion or a recent COVID‐19 infection. Severe orthopnea, diaphoresis, cool extremities, tachycardia, pulsus paradoxus, a low PI, and a progressive pericardial effusion on serial imaging collectively suggested evolving tamponade physiology, with systemic perfusion likely maintained by compensatory tachycardia and sympathetic activation.

The educational value of this case is not that pericardial drainage was performed for cardiac tamponade; such drainage is an established treatment when tamponade physiology is present [1, 4, 5]. Rather, its value lies in the decision to defer the planned induction of general anesthesia, perform a rapid reassessment, and obtain urgent multidisciplinary input before proceeding. Because anesthetic drugs and positive‐pressure ventilation may reduce sympathetic tone, venous return, and cardiac filling, proceeding with induction under the initial working diagnosis could have precipitated hemodynamic collapse [1, 4, 6]. The anesthesia team's independent review and decision to defer induction enabled pre‐induction stabilization under local anesthesia, a strategy consistent with the broader principle of stabilizing obstructive or preload‐dependent cardiovascular physiology before induction whenever feasible [2, 3].

Echocardiography plays a central role in assessing the hemodynamic significance of pericardial effusion. Echocardiographic findings supporting tamponade physiology include right atrial and right ventricular diastolic collapse and exaggerated respiratory variation in mitral and tricuspid inflow velocities, reflecting enhanced ventricular interdependence [7]. These findings should be assessed systematically when cardiac tamponade is suspected. In retrospect, the lack of a repeat echocardiographic assessment before transferring the patient to the operating room was a major limitation of the initial evaluation. Serial CT had shown a progressive pericardial effusion, but CT alone cannot determine the hemodynamic effects of a pericardial effusion. An earlier multidisciplinary discussion and a repeat echocardiographic assessment may have identified the evolving tamponade physiology before the patient's transfer. Nevertheless, once the patient arrived in the operating room with signs of shock and orthopnea, the anesthesia team's decision to defer induction, establish invasive monitoring, and obtain an urgent multidisciplinary consultation enabled pre‐induction stabilization and averted potential hemodynamic collapse. Ideally, such a patient should be identified earlier and managed in a monitored setting (e.g., intensive care unit [ICU]) before being transferred to the operating room. In reality, however, anesthesiologists may occasionally encounter patients in whom the preoperative evaluation is incomplete and clinical deterioration becomes evident immediately before induction; our case illustrates a practical approach to rapid reassessment, escalation, and pre‐induction stabilization in this setting.

In this case, PI was interpreted only as one of several immediately available bedside warning signs, not as a diagnostic substitute for echocardiography or point‐of‐care ultrasound (POCUS) [8]. No single numeric threshold was used to determine the need for pericardial drainage. Rather, the decision was based on the convergence of severe orthopnea, diaphoresis, cool extremities, tachycardia, respiratory variation in the arterial pressure waveform, a low PI, and a progressive pericardial effusion on imaging. This case emphasizes that continuously available monitoring signals may prompt an urgent reassessment, but echocardiography remains central to evaluating the hemodynamic significance of a pericardial effusion [7].

This case highlights the importance of independent pre‐induction reassessment when a patient's clinical presentation upon arrival in the operating room is discordant with the initial working diagnosis. In a patient with a previously recognized but clinically underestimated pericardial effusion, severe orthopnea, shock‐like bedside findings, respiratory variation in the arterial pressure waveform, and a low PI prompted the decision to defer induction and obtain urgent multidisciplinary reassessment. Emergency cases with incomplete or evolving preoperative information require a low threshold to defer induction and reassess cardiovascular status before proceeding with general anesthesia.

## Author Contributions

**Itsuki Suganuma:** data curation, writing – original draft, writing – review and editing. **Tomoo Yuba:** conceptualization, data curation, formal analysis, writing – original draft, writing – review and editing. **Katsunao Tanaka:** writing – review and editing, supervision. **Yu Matsumoto:** supervision, writing – review and editing. **Akira Iura:** supervision, writing – review and editing, funding acquisition. **Takeshi Yoshida:** supervision, writing – review and editing.

## Funding

This work was supported by JSPS KAKENHI (Grant number 26K11834).

## Consent

Written informed consent was obtained from the patient for publication of this case report and accompanying images. A copy of the written consent is available for review by the Editor‐in‐Chief of this journal.

## Conflicts of Interest

The authors declare no conflicts of interest.

## Data Availability

Data sharing is not applicable to this article as no datasets were generated or analyzed beyond the clinical information presented in this case report.
